# Quantitative MRCP metrics as imaging biomarkers to differentiate benign from malignant bile duct obstructions

**DOI:** 10.3389/fonc.2025.1576163

**Published:** 2025-05-06

**Authors:** Kulyada Eurboonyanun, Julaluck Promsorn, Prakasit Sa-Ngiamwibool, Chalerm Eurboonyanun, Sarah Finnegan, Carlos Ferreira, Amy Herlihy, Elizabeth Shumbayawonda, Rita Maria Lahoud, Isha Atre, Aileen O’Shea, Mukesh Harisinghani

**Affiliations:** ^1^ Department of Radiology, Faculty of Medicine, Khon Kaen University, , Khon Kaen, Thailand; ^2^ Department of Pathology, Faculty of Medicine, Khon Kaen University, Khon Kaen, Thailand; ^3^ Department of Surgery, Faculty of Medicine, Khon Kaen University, Khon Kaen, Thailand; ^4^ Translational Science, Perspectum Ltd, Oxford, United Kingdom; ^5^ Department of Radiology, Tufts Medical Center, Boston, MA, United States; ^6^ Department of Radiology, Children’s Hospital of Pittsburgh, Pittsburgh, PA, United States; ^7^ Radiology Department, Massachusetts General Hospital, Boston, MA, United States

**Keywords:** cholangiocarcinoma, bile duct, biliary obstruction, MRCP+, early diagnosis

## Abstract

**Background:**

Cholangiocarcinoma (CCA) is a difficult-to-detect rare cancer with high mortality rate and management costs. If detected early, surgical resection carries a 35% 5-year survival rate; this decreases to <11% 1-year survival rate when detected at later stages. Quantitative magnetic resonance cholangiopancreatography (MRCP+) provides measurements of the biliary tree and has been noted in clinical guidelines as having prognostic utility. We sought to determine whether MRCP+ metrics could differentiate benign and malignant biliary obstructions.

**Method:**

In this retrospective study of 38 patients with biliary obstruction with histologic characterisation, 23 had malignant obstructions whilst 15 had benign obstructions. Patients underwent non-contrast and contrast MRCP alongside clinical assessment. Non-contrast MRCP images were post-processed with MRCP+. Mann-Whitney U test compared the metrics between groups. Diagnostic accuracy of MRCP+ markers (duct number and dimensions, biliary tree and gallbladder volume) to stratify benign from malignant biliary obstructions was assessed using the area under the receiver operating characteristic curve (AUC).

**Results:**

All bile duct metrics were significantly higher in malignant biliary obstruction (p<0.05). Of the metrics assessed, total biliary tree volume was the most clinically meaningful predictor of malignancy, with a volume of ≥25ml differentiating between the two populations. A biliary tree volume of 25ml had an AUC of 0.79 to stratify between benign and malignant obstructions.

**Conclusion:**

Quantitative MRCP metrics, particularly total biliary tree volume, are shown here to differentiate malignant (CCA) from benign obstructions. As current pathways require either contrast administration or ERCP, quantitative MRCP may be an objective, non-invasive tool to identify CCA.

## Background

Biliary obstructions, such as those observed with both benign and malignant cancers, are an increasing cause of concern as late detection is associated with high morbidity and mortality (1). Biliary dilatation, frequently encountered in clinical practice, can be idiopathic, caused by various obstructive or non-obstructive diseases (2) due to anatomical variants (3).The most common cause of all biliary obstructions are periampullary cancers, with a reported incidence of 37.2 to 72.5% in those with suspected pathology (2, 4). Benign obstructions are either secondary to choledocholithiasis or are associated with other conditions such as inflammation, infection, or ischemia (2, 5). Regarding epidemiology and risk of benign obstructions, race and sex (male vs female) play a key role. For instance, in the case of choledocholithiasis which affects ~6% of the global population, those of Hispanic and Northern European ethnicity are at higher risk compared to those of Asian or African ethnicity (6). Furthermore, women are more likely to develop gallstones and gallbladder cancer compared to males (7). The treatment of biliary dilatation varies depending on the cause. For malignant obstructive lesions, surgery is the primary curative treatment (2), whilst non-obstructive dilatation might require no treatment or can be treated with minimally invasive procedures like endoscopic dilatation or stent insertion (8). For instance, when detected at early-stage and curative surgery performed, cholangiocarcinoma (CCA) is associated with a 35% 5-year survival rate compared with less than 12 months when detected at late stage (9). Thus, for patients to receive appropriate management in a timely manner, radiologists and clinical practitioners need to distinguish malignant- from non-malignant obstructions. For instance, although cholangiocarcinoma affects fewer than 2 individuals per 100,000, managing the condition costs ~US$14,000 per patient per month (10, 11) and is associated with nearly 20% of hepatobiliary cancer deaths (12). Similarly, benign obstructions, like choledocholithiasis, account for ~US$10,000 per patient depending on the severity and management required (13). Nevertheless, despite these healthcare costs, due to the challenges associated with differentiating benign from malignant biliary obstruction using traditional methods, the detection of CCA remains a challenge (14).

Generally, bile duct dilatation is defined as a common bile duct (CBD) with a maximal diameter exceeding 6 mm (15) however, there is a significant overlap between normal and dilated bile duct diameters (16). For instance, in those aged 65 years and younger, normal CBD diameters can be up to 8 mm, whilst those older than 65 years of age have normal CBD diameters of up to 11 mm (17). Not only age but factors such as prior cholecystectomy can affect CBD diameter in otherwise healthy individuals (18, 19). Furthermore, the biliary tree is not the same size throughout even in healthy individuals, and, in some cases, pathological dilatation (be that benign or malignant) is not uniform throughout the tree which further complicates diagnosis. Although some literature suggests using different thresholds for different parts of the biliary tree, this approach is complicated and has yet to be widely adopted and used in clinical practice (20).

Apart from differentiating a pathologically dilated bile duct from a normal bile duct, there is also difficulty in determining whether the pathologically dilated duct is secondary to a benign or malignant cause. Previous studies showed that magnetic resonance imaging (MRI) has high sensitivity and specificity for differentiating benign from malignant biliary obstruction (21). Bile ducts with malignant obstruction tend to be irregular and asymmetric, have greater dilatation, involve more extended segments, and are associated with wall thickening and enhancement (8, 9). However, benign conditions can sometimes also manifest as irregular or asymmetric strictures, creating a clinical challenge and occasionally leading to repeated investigations or unnecessary procedures (22–24). In addition to the use of MRI, there are several modalities available for imaging the biliary tree with varying ranges of invasiveness, including ultrasonography (US), computed tomography (CT), magnetic resonance cholangiopancreatography (MRCP), endoscopic US (EUS), and endoscopic retrograde cholangiopancreatography (ERCP). However, although ERCP is a reference standard for diagnosis of CCA, outperforming US, CT and EUS and allowing for intervention, it is invasive and associated with increased likelihood of complications including pancreatitis, perforation, and bleeding (25). MRCP allows detailed, non-invasive evaluation of the biliary tree without exposure to ionizing radiation or procedure-related complications, and it is generally accepted as a replacement for ERCP for the diagnosis of biliary pathology (26). Additionally, MRCP can be used as an assistive tool to identify those who would benefit from a further therapeutic ERCP or EUS procedure (20). However, similar to the majority of currently used techniques, MRCP is a subjective method and thus suffers from inter-reader variability which may result in delayed therapeutic intervention in patients with a high clinical suspicion of bile duct obstruction (27).

Quantitative MRCP (MRCP+) is an artificial intelligence-enabled (AI-enabled) software that enhances conventional MRCP to produce quantitative MRCP models which provide metrics that accurately and reliably characterise the biliary tree (28, 29), supporting both visualisation and direct assessment of ductal anatomy without additional scan time or contrast. In addition to being noted in the EASL PSC guidelines as having utility as a prognostic tool for prediction of clinical outcomes in primary sclerosing cholangitis (PSC) (30), MRCP+ metrics have shown utility to support risk stratification in PSC (31), to stratify autoimmune hepatitis (AIH) from AIH/PSC overlap (32, 33), and to be independent predictors of risk outperforming the MAYO and SCOPE risk scores (34). MRCP+ metrics have also been shown to correlate well with markers of disease progression alongside correlating with prognostic factors in PSC (28, 35). With the growing body of evidence highlighting the clinical utility of MRCP+, we sought to explore the usage of MRCP+ beyond the scope of the PSC population. Recently, a study investigating the utility of direct access and MRCP+ in the assessment of suspected acute biliary or ductal gallstone presentations found that those with acute gallstone disease had higher biliary tree and gallbladder volumes compared to those without (36). Building on these results highlighting the potential utility of using automated biliary tree measurements for detecting biliary obstruction, our study aimed to extend these findings by evaluating whether objective, non-invasive metrics derived from MRCP+ can effectively stratify different obstruction aetiologies. Specifically, we sought to determine the ability of MRCP+ metrics to identify the underlying causes of biliary obstruction in non-PSC patients.

## Methods

### Study population

Patients with abnormal bile duct dilatation who underwent MRI/MRCP and further endoscopic or surgical procedures from March 2013 to March 2016 were included in the study. Clinical data (including age, gender, presenting symptoms, and laboratory results) were collected from the electronic medical records. The MRI and MRCP images were retrieved from the Picture Archiving and Communication System (PACS) (SYNAPSE, Fujifilm Medical Systems USA, Inc.).

### Ethical consideration

This was a single centre study which received ethical approval from the institutional (Massachusetts General Hospital) Mass General Brigham review board (ethics reference: HE631095). All enrolled participants gave written informed consent to participate in the study. The principles identified in the 1975 Declaration of Helsinki and GCP principles were observed throughout the study. All participant-identifiable information was kept securely and encrypted within the servers at the study site.

### Imaging protocol and post-processing

Both non-contrast and contrast enhanced MRIs of the upper abdomen and MRCP images were acquired using either a 1.5T Aera Siemens scanner (Siemens Healthineers, Erlangen, Germany) or 3.0T Achieva Philips scanner (Philips Healthcare, Massachusetts, USA) using a standard protocol (**Supplementary Table 1**). All images were collected in the same scanning session. The MRCP images were obtained using 3D multi-shot fast/turbo spin echo acquisitions, with very long echo train lengths and short echo spacing, to generate heavily T2-weighted three-dimensional volumetric images. Seventy-two contiguous slices were acquired with a field of view of 400 x 400, an acquisition matrix of 258 x 320, and a reconstruction matrix of 320 x 320, resulting in a voxel resolution of 1.25 x 1.1 x 1.25 mm for all scans. Data was acquired with respiratory gating (using navigator tracking) and during the expiration phase, so that the repetition time (TR) varied with breathing rate. Fat suppression techniques were used to suppress signal from fat, and parallel imaging techniques to reduce scanning time.

**Figure 1:**
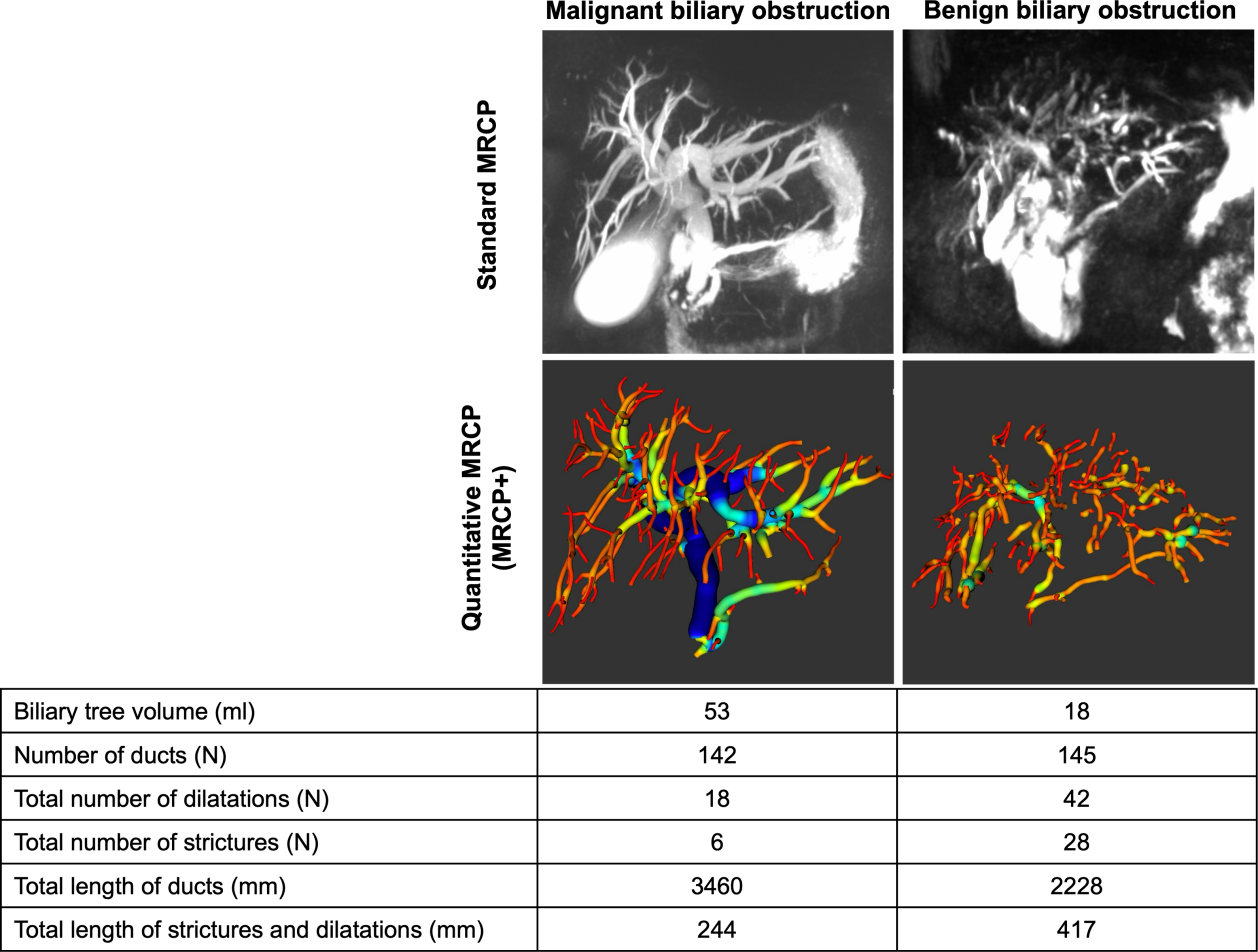
MRCP+ models for two patients diagnosed with either benign or malignant biliary obstructions.

Following acquisition and de-identification according to HIPAA standards, non-contrast MRCP images were post processed using MRCP+ (Perspectum Ltd., United Kingdom) to generate the biliary tree metrics (**Figure 1**). During post-processing, in addition to checking the image quality (including slice thickness, orientation, voxel resolution, signal-to-noise ratio, gastrointestinal contamination), a colour-coded 3D model of the biliary tree showing the variation in diameter along each duct was generated (29). Two experienced abdominal radiologists (with >15 years’ experience each) retrospectively reviewed the T2-weighted images (T2WI), diffusion-weighted images and corresponding apparent diffusion coefficient map (DWI/ADC), and dynamic contrast enhancement (DCE) MR images. It is worth nothing that the in addition to being able to analyse standard 3D MRCP images, there is an MRCP+ imaging protocol (Perspectum Ltd., United Kingdom) which is standardised across both scanner and (Siemens, Philips, GE) and field strength (1.5T and 3T) (28, 29).

### Bile duct obstruction definition: benign or malignant

The radiologists determined the above findings (a combination of mass, abnormal restriction, level and length of obstruction, and ductal wall morphology such as mural thickening or luminal irregularity using all MRI sequences) and subjectively decided the aetiology of the obstruction. To assess the aetiology of the obstruction, the two expert readers used a combination of mass, abnormal restriction, level and length of obstruction, and ductal wall morphology such as mural thickening or luminal irregularity using all MRI sequences including T1WI, T2WI, MRCP, DWI, and post-contrast images. The readers were blinded to all clinical data including pathology and laboratory results. Each radiologist independently reviewed the images and gave an overall impression of whether the obstruction was benign or malignant. For assessing intra-observer agreement, each radiologist performed two imaging sessions separately. Each imaging session was at least two weeks apart to minimize recognition bias. Furthermore, radiologists were blinded to the MRCP+ metrics.

### Inclusion and exclusion criteria

As this was a retrospective study, inclusion criteria included consecutive patients who underwent MRCP and subsequently had a biopsy or resection. To ensure sufficient follow-up time for benign cases and rule out the possibility of false negatives, patient data were collected between 2020 to 2021. Exclusion criteria were limited to those where choledocholithiasis was visible in MRCP images and those whose MRCP images were of insufficient quality for analysis. All patients included underwent either endoscopic biopsy or open surgery and final diagnosis was based on histopathological assessment.

### Statistical analysis

Descriptive statistics were used to summarise baseline participant characteristics. Categorical data were reported using numbers and percentages whilst continuous data were reported using means with standard deviation (± SD) or with interquartile ranges (IQR).

Comparison of MRCP+ metrics between those with benign and malignant biliary obstructions were performed using nonparametric tests. Univariate logistic regression models were fitted to assess the diagnostic performance of individual imaging predictors (MRCP+ metrics) to stratify between malignant and benign biliary obstructions. Receiver operating characteristic (ROC) curves were generated and area under the ROC curve (AUC) as well as its 95% CI was estimated. Youden’s index was used to calculate an ideal cut-off, with the associated sensitivity, specificity, negative prediction value (NPV), positive prediction value (PPV) and accuracy determined.

To further assess added benefit and utility of using MRCP+, patients were divided into two groups (group1: mid- to upper CBD-, perihilar, or intrahepatic obstruction vs. group 2: distal CBD and multifocal obstructions) and the performance of MRCP+ to the expert readers was compared using AUC.

Inter- and intra-reader variability between the radiologists’ readers and MRCP+ cut-off were assessed using Cohen’s Kappa (Kappa, ĸ). During statistical analyses, potential confounders such as age, sex, and previous cholecystectomy were not controlled for. All statistical analyses were performed using IBM SPSS Statistics for Macintosh, version 22.0 (Armonk, NY: IBM Corp.), and values of p<0.05 were considered statistically significant.

## Results

### Patient demographics and diagnosis

In this study, N=46 patients met the inclusion criteria after which N=8 were excluded due to technical imaging problems incurred during MRCP acquisition (insufficient image resolution resulting in poor quality MRCP). The N=38 who were included in the final analysis had mean age 65 ± 10 years and 66% were male. **Table 1** shows a summary of patient demographics. Of the 38 patients, N=18 underwent resection.

**Table 1:** Patient demographics and radiology assessments.

	Benign (N =15)	Malignant (N = 23)	Available N	p-value
Patient Demographics				
Sex (Male)	11	13	38	0.542
Age	67 ± 11	61 ± 15	38	0.157
Laboratory Investigations				
Liver Function Tests				
Cholesterol	146.50 (176.00 - 207.00)	166.00 (116.00 - 207.00)	25	0.461
Total Protein	6.80 (5.95 - 7.30)	7.10 (6.20 - 8.60)	25	0.196
Albumin	3.70 (2.93 - 4.15)	3.70 (3.20 - 4.00)	25	0.765
Total Bilirubin	0.70 (0.575 - 5.950)	1.60 (0.475 - 4.900)	24	0.977
Direct Bilirubin	0.45 (0.200 - 3.750)	0.95 (0.200 - 3.575)	24	0.625
ALT	34.00 (21.75 - 106.50)	32.00 (22.00 - 63.00)	25	0.935
AST	42.50 (28.00 - 179.00)	43.00 (36.00 - 96.00)	25	0.807
ALP	128.00 (77.75 - 197.25)	148.00 (119.00 - 217.00)	25	0.428
PT	12.40 (10.40 - 14.30)	13.85 (11.15 - 14.525)	17	0.541
INR	1.19 (1.05 - 1.335)	1.29 (1.045 - 1.3725)	17	0.606
Tumor Markers				
CA19-9 (U/mL) (reference range: <37 U/mL)	8.03 (1.18 - 13.00)	14.93 (1.66 - 30.74)	18	0.375
CEA (ng/mL) (reference range: <2.5 ng/mL)	2.78 (2.26 - 6.97)	1.93 (1.58 - 3.72)	15	0.463
AFP (ng/mL) (reference range: <10 ng/mL)	2.09 (0.96 - 68.40)	2.87 (2.06 - 3.72)	10	0.730
Radiologist Assessment				
Reader 1: Number correctly identified				
Read 1	13	18	38	**<0.001**
Read 2	13	20	38	**<0.001**
Reader 2: Number correctly identified				
Read 1	13	22	38	**<0.001**
Read 2	15	21	38	**<0.001**

All p values <0.05 are indicated in bold.

Histopathology assessment showed that N=23 patients (58%) were diagnosed as having malignant conditions with N=15 (39%) having cholangiocarcinoma, N=6 (16%) intraductal papillary neoplasm of the bile duct (IPNB), and N=2 (5%) having ampullary adenocarcinoma. Of the N=15 (39%) diagnosed with benign conditions, N=13 (34%) had benign strictures with associated inflammation and fibrosis and N=2 (5%) had neoplasms (ampullary adenoma and CBD tubulovillous adenoma). In this cohort, there were no significant differences in the incidence of malignancy between male and female patients (52% vs. 69%) (p=0.490). Furthermore, the mean age of patients with benign and malignant obstructions was not statistically significant (68 ± 11 years for benign disease vs. 63 ± 8 years for malignant disease) (p=0.171) (**Table 1**). **Figure 1** shows an illustration of MRCP+ models for a patient with benign and another with malignant obstructions. In this cohort, N=15 (39%) underwent cholecystectomy prior to inclusion.

Among patients with available laboratory investigation, there were no significant differences in the liver function test (serum cholesterol, total serum protein, total serum albumin, total- and direct bilirubin, aspartate transferase (AST), alanine transaminase (ALT), alkaline phosphatase (ALP), and prothrombin time (PT/INR)) between patients with benign and malignant obstructions. Additionally, the tumour markers, available in a smaller portion of patients, also showed no significant difference in both groups (**Table 1**). There were only three patients with cancer antigen 19-9 (CA19-9) levels above 37 ng/mL. Furthermore, only 2 out of 11 patients (18.2%) with malignant obstruction had abnormal CA19–9 levels. The proportions of patients with elevated serum CA 19–9 in benign and malignant group were not significantly different (p = 1.000).

### Biliary obstruction level and surgical intervention

As prior cholecystectomy can affect CBD diameter, we investigated the differences between bile duct volume cholecystectomy- (N=15) and non-cholecystectomy patients. No significant differences (p=0.464) were identified between the groups (30.50 (16.7 – 57.6) ml. vs. 38.7 (8.8 – 86.0) ml respectively).

Regarding the biliary obstruction level, 18 patients (47%) had distal CBD obstruction, 10 (26%) had perihilar obstruction, 4 (11%) had intrahepatic duct obstruction, 2 (5%) had mid-upper CBD obstruction and 2 (5%) had multifocal obstructions. We performed a subgroup analysis by dividing the patients into two groups: group 1 – patients with mid- to upper CBD, perihilar, or intrahepatic obstructions, and group 2 – patients with distal CBD and multifocal obstructions. This resulted in 18 patients in group 1 (all malignant) and 20 patients in group 2 (15 benign and 5 malignant). Comparisons between the groups showed that group 1 had a significantly higher biliary volume than group 2 (median 40.8 ml [range 24.2–65.7 ml] vs 26.2 ml [range 10.9–72.5 ml], p < 0.001 respectively). Within group 2, the biliary volume in patients with malignant obstructions was significantly higher than in those with benign obstructions (median 67.2 ml [range 45.7–125.0 ml] vs 16.7 ml [range 7.9–25.3 ml], p = 0.016, respectively).

### Associations between imaging markers and biliary obstructions

Compared with benign obstruction, patients with malignant biliary obstruction had significantly higher values of almost all available MRCP+ metrics except the gallbladder volume (**Table 2**). Those with malignant biliary obstructions had higher biliary volume (p = 0.001), number of ducts (p = 0.038) and total length of ducts (p = 0.002). Moreover, those with benign biliary obstructions had significantly lower metrics relating to severity of pathology including total number of strictures (p = 0.008), total number of dilatations (p = 0.001) and the total length of strictures and dilatations (p = 0.001) (**Table 2**).

**Table 2:** Groupwise differences in quantitative MRCP metrics between those diagnosed with either benign or malignant biliary obstructions.

	Benign (N=15)	Malignant (N=23)	p-value
Biliary tree volume (ml)	18.2 (16-56)	40.7 (26-58)	**0.001**
Number of ducts	145 (64-277)	164 (129-190)	**0.038**
Total number of candidate strictures	16 (12-27)	17.5 (12-21)	**0.008**
Total number of candidate dilatations	33 (23-49)	38.5 (26-54)	**0.001**
Total length of ducts (mm)	2228 (1353-5450)	3278.4 (2489-4389)	**0.002**
Total length of candidate strictures and dilatation (mm)	253 (79-330)	629.15 (346-933)	**0.001**
Gallbladder volume (ml)	32.3 (13-68)	42.75 (24-66)	0.224

All p values <0.05 are indicated in bold.

### Concordance with radiologist assessments

Following histopathological assessment, both radiologists assessed the MRCP images and could discriminate benign from malignant lesions (p<0.001). In both reads, both readers had high intra-reader agreement (reader 1: ĸ = 0.788 and reader 2: ĸ = 0.838) for the identification of biliary obstruction type (**Table 1**). Additionally, the inter-observer reliability ranged from substantial to excellent (ĸ = 0.680 to ĸ = 0.839).

When comparing the ability of biliary tree volume derived by MRCP+ to differentiate benign from malignant obstruction and the radiologists’ performance, MRCP+ performance was similar to that of the expert readers. More specifically, when looking at the cases in group1 (mid- to upper CBD-, perihilar, or intrahepatic obstruction) (N=18, all malignant), using a volume of 25ml missed 3 malignant cases which is comparable to both reader 1 (3 missed malignant cases in the first read, 2 in the second read), and reader 2 (1 missed case in the first read, 2 in the second read). Looking at the cases in group 2 (distal CBD and multifocal obstructions), a biliary volume of 25ml could identify N=16 (11/15 benign and 5/5 malignant) obstructions. In this group, reader 1 identified N=16 obstructions (13/15 benign and 3/5 malignant) in read 1 (N=17 in read 2; 13/15 benign, 4/5 malignant). Reader 2 identified N=18 obstructions (13/15 benign and 5/5 malignant) in read 1 and all cases correctly in read 2. MRCP+ was found to perform similarly to expert readers in the identification of lesions with no significant differences identified in performance (p = 0.2234).

### Predictive capability of liver biochemistry and quantitative imaging

**Figure 2:**
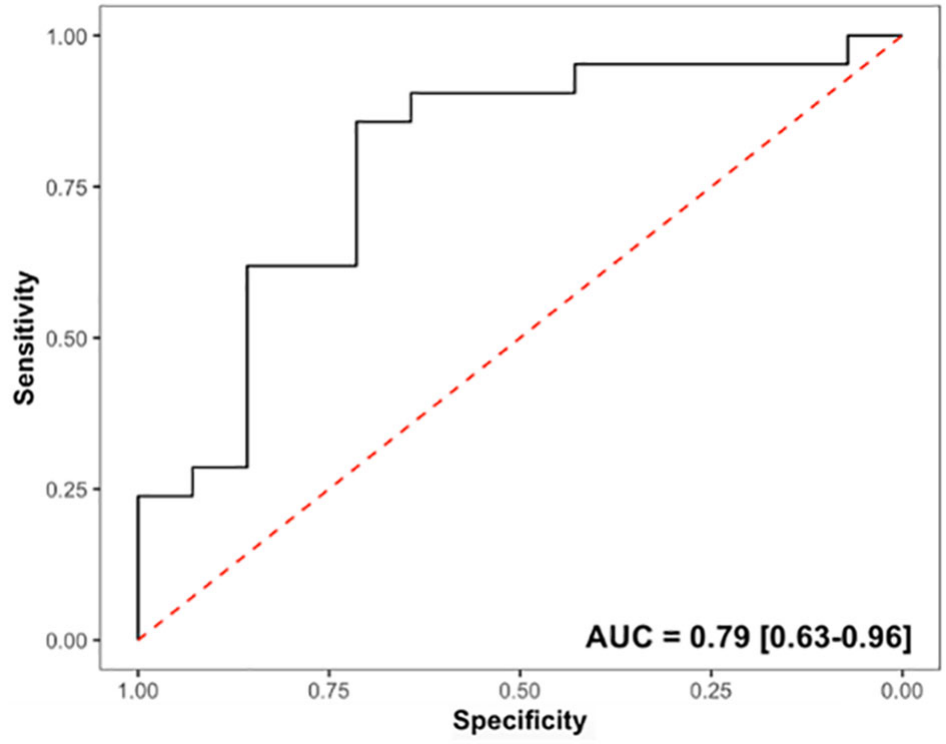
Diagnostic accuracy of biliary tree volume to stratify between patients with benign or malignant biliary obstructions.

ROC analyses were performed to assess the capability of MRCP+ imaging metrics to distinguish between malignant and benign biliary obstructions, the results of which are shown in **Table 3**. MRCP+ metrics also showed very good diagnostic ability to stratify between biliary obstruction type with biliary tree volume having an AUC of 0.79 (95% CI: 0.63 – 0.96) with a Youden’s cut-off of 25.4 ml (**Figure 2**, **Supplementary Figure 1**). Median biliary tree volume of those with malignant biliary obstructions was triple that of those with benign strictures (53.10 ml [IQR 30.50-84.60] vs 16.70 ml [IQR 7.90-25.30], p = 0.001).

**Table 3:** Assessment of the diagnostic performance of imaging parameters for discriminating malignant from benign biliary obstructions using area under the receiver operating characteristic (AUC) curve analyses.

	AUC	95% CI	Cut-off	Sensitivity	Specificity	PPV	NPV	p-value
Biliary tree volume	0.79	0.63-0.96	25	0.86	0.64	0.78	0.75	**0.0015**
Number of ducts	0.77	0.59-0.95	124	0.86	0.71	0.82	0.77	**0.0041**
Total number of strictures	0.76	0.58-0.94	12	0.86	0.57	0.75	0.73	**0.0052**
Total length of ducts	0.8	0.63-0.97	2756	0.81	0.79	0.85	0.73	**0.0012**
Total length of strictures and dilatation	0.81	0.66-0.96	338	0.81	0.79	0.85	0.73	**0.00081**
Total number of dilatations	0.81	0.66-0.96	26	0.86	0.71	0.82	0.77	**0.001**
Gallbladder volume	0.58	0.39-0.78	12	0.62	0.64	0.72	0.53	0.2

All p values <0.05 are indicated in bold.

**Figure 3:**
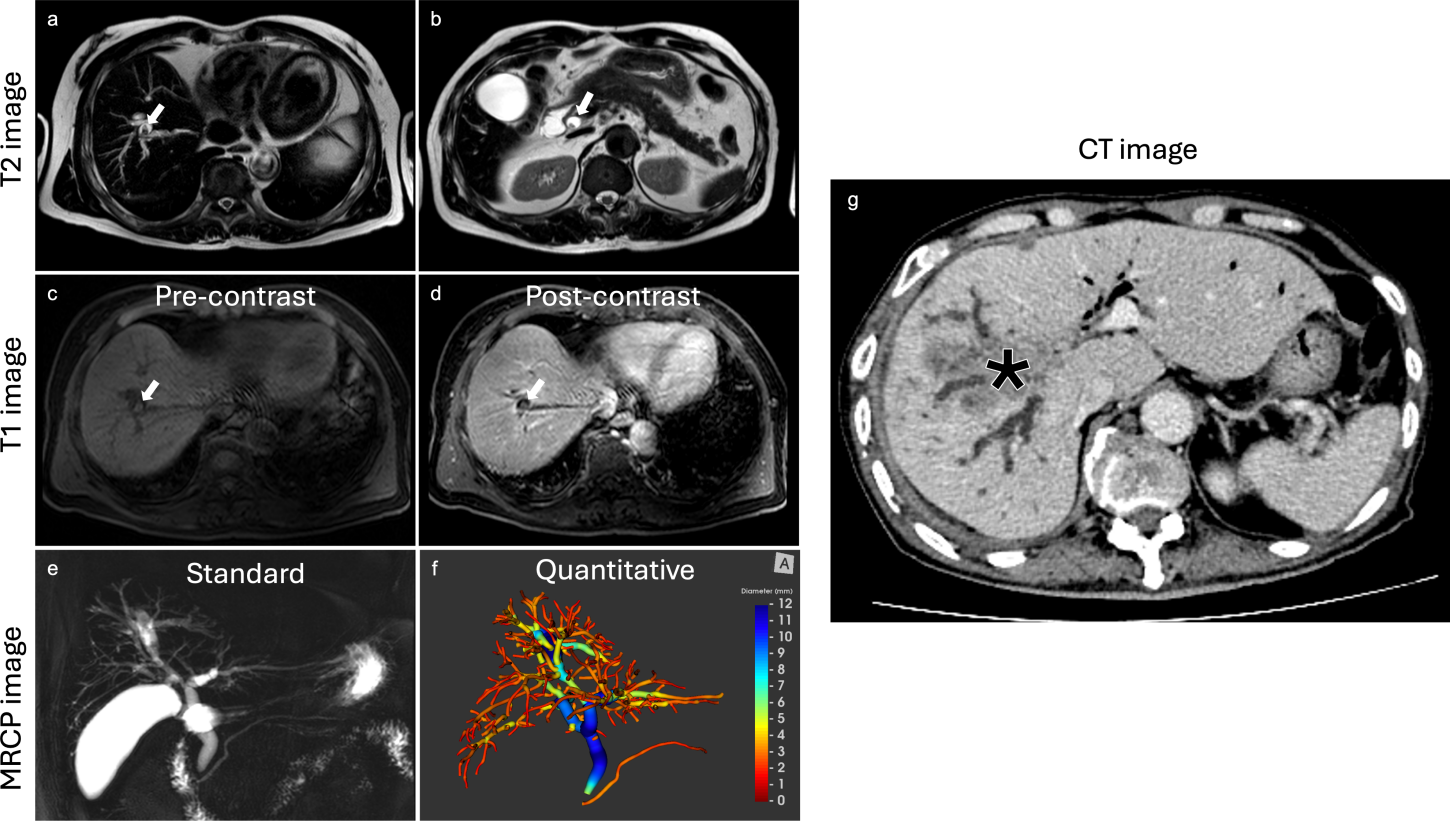
Case example highlighting the clinical utility of MRCP+. In this case, a lesion was initially diagnosed as benign on histology and reported by radiologists as being benign using T2 T1 and MRCP images. The biliary tree volume for this case was 43.8ml. The lesion progressed to cholangiocarcinoma eight years after as shown by the CT image. **(a)** axial T2 image revealing non-enhancing stones highlighted by white arrow, **(b)** axial T2 image with visible non-enhancing stones highlighted by white arrow, **(c)** axial T1 image with fat suppression exhibiting non-enhancing stones highlighted by white arrow, **(d)** axial T1 post-contrast image with fat suppression demonstrating non-enhancing stones highlighted by white arrow, **(e)** standard MRCP image showing diffuse dilatation of intrahepatic and extrahepatic bile ducts, **(f)** MRCP+ model with high biliary tree volume, all at baseline. After eight years, **(g)** axial follow-up CT image revealing an intrahepatic cholangiocarcinoma (asterisk).

Using a biliary tree volume threshold of 25 ml or more as a threshold, 20/23 (86.4%) malignant obstruction and 11/15 (68.8%) benign obstruction were correctly identified. Thus, a biliary tree volume of 25 ml had sensitivity: 86.96%, specificity: 73.33%, NPV: 78.57%, PPV: 83.33%, and accuracy: 81.58% to identify biliary obstruction type. **Figure 3** illustrates the utility of MRCP+ to identify subtle early changes in biliary tree volume which can be linked to progression to CCA.

## Discussion

In this study we sought to determine whether quantitative MRCP metrics could be used to differentiate benign and malignant biliary obstructions. Our findings showed that MRCP+ metrics, in particular total biliary tree volume of 25ml (AUC: 0.79, sensitivity: 0.86, specificity: 0.64), have good diagnostic performance to differentiate malignant biliary obstructions (majority of which were CCA) from benign biliary obstructions. Given that current patient pathways require either contrast administration or ERCP (a procedure with associated increased risks of mortality and morbidity) to differentiate between the two, quantitative MRCP metrics may offer an objective, non-invasive biomarker to identify malignant biliary obstructions such as CCA.

Biliary dilatation is not an uncommon finding, with over two-thirds of cases due to malignant biliary obstructions. Delayed diagnosis, especially in the case of malignant obstructions, can result in significant morbidity and mortality (37). Cholelithiasis, the most common cause of benign biliary tract obstruction, can be reliably diagnosed with MRCP (38, 39). However, discrimination between malignant and benign biliary obstruction from causes other than cholelithiasis is more challenging. Several studies have reported on MRI’s ability to differentiate benign and malignant biliary obstruction, with evidence showing significant correlation between MRCP and ERCP findings with histological or surgical outcomes (21, 40–43). However, although metrics such as hyperintensity on diffusion weighted imaging (DWI), lower apparent diffusion coefficient (ADC) value, thick bile duct wall, longer segment involvement, hyperenhancement of the bile duct, luminal irregularity, and asymmetry of strictured bile duct (42–44) have shown good performance, these features are either subjective or require interpretation from experienced radiologists, thus making it difficult to reproduce or compare the studies across different institutions.

Quantitative MRCP imaging, an accurate and repeatable assessment of the biliary tree, provides detailed information about the number, length, and severity of strictures and dilatations, as well as the total volume of the biliary tree (29). By providing a 3D model of the biliary system, MRCP+ enables the measurement of bile duct widths and automatic detection of regions of variation of duct widths thereby allowing for regional volumetric analysis of the biliary tree, pancreatic duct, and gallbladder using non-contract MRCP images (29). In this study, MRCP+ metrics quantifying biliary anatomy (biliary volume, number of ducts, total length of ducts) and severity of pathology (total number of strictures, total number of dilatations, the total length of strictures and dilatations) had good diagnostic performance in stratifying between benign and malignant biliary obstructions. We found that using a biliary tree volume ≥ 25 ml had good utility to stratify between benign or malignant obstructions with accuracy comparable to experienced radiologist. This is particularly important as typically radiologists must review images from many MR sequences, including traditional T1WI, T2WI, DWI/ADC, thin-and-thick slab MRCP, and DCE, to make a diagnosis. Therefore, in addition to the time taken to review images, the time to diagnosis can be delayed and result in development of adverse clinical outcomes. MRCP+ has clinical utility to support patient management as a screening tool in those with suspected malignant biliary obstructions as it requires only non-contrast 3D-heavily-T2-weighted MRCP images which are already acquired as part of a patient’s standard of care, thereby significantly reducing scan time and resource use. Furthermore, as MRCP+ is noninvasive, its use avoids the adverse effect associated with ERCP and thus, can be used as part of standard-of-care as a test to support patient triage prior to potential ERCP. Considering the heterogeneity of bile duct adaptations observed in different biliary disease states, the adoption of quantitative evaluation of MRCP images has the potential to improve diagnostic performance, reduce clinician burden and sensitively monitor ductal change over time (45). For cases with indeterminate biliary strictures (IDBS), as MRCP+ provides quantitative metrics which can be monitored over time, it can be used as part of the follow-up assessments to monitor changes in the biliary tree over time. Similar to that shown in PSC risk prediction, future studies should look at investigating the utility of MRCP+ metrics in this population to support early identification of patients requiring intervention (24).

Lastly, there are several tumour markers for malignant hepatobiliary lesions, such as CA 19-9, carcinoembryonic antigen (CEA), and cancer antigen 125 (CA 125) (46). CA 19-9, the most clinically established biomarker for cholangiocarcinoma screening, is increasingly used for differential diagnosis of benign and malignant hepatobiliary conditions (46, 47). However, its diagnostic power is currently limited (48–50). Our study, though small in scale, demonstrated the potential of quantitative MRCP, showing its superiority over conventional serum biomarkers and paving the way for its future role in diagnosis.

There were some limitations to our study. First, our study only included patients with biopsy proven diagnoses. Therefore, it is possible that our results may be bias as patients with benign obstruction who received interval follow-up without endoscopic intervention were not included. However, as patients with suspected malignancy typically undergo biopsy (current diagnosis reference standard), to ensure that histopathological assessment was included in our clinical assessment we restricted inclusion to only those patients with proven diagnosis on biopsy. Both radiologists included in this study are experts with significant hepatobiliary imaging experience from a tertiary care hospital with a high volume of hepatobiliary cases. Therefore, it is possible that these findings may vary across centres. Nevertheless, as subjectivity and expertise vary across centres, the use of standardised objective tools such as MRCP+ could support standardisation of clinical assessment of the biliary tree. This was a cross-sectional study, and evaluation of the utility of MRCP+ markers to both monitor disease progression/regression was not performed. As MRCP+ is an objective tool ideally suited for long-term monitoring, future studies looking at longitudinal assessment will yield a better understanding of the changes associated with these metrics and thus will reveal the added impact these metrics have on monitoring of disease progression over time, the sensitivity of the metrics to change, and their associations with important clinical outcomes. Last, although multiple studies have shown the prevalence of malignant cancers to be similar to that reported here (39), this was a real-world study with a relatively small cohort of patients with obstructions. Future studies should validate these findings in larger cohorts and investigate if the combination of MRCP+ metrics with blood markers will enhance classification. This is particularly so as some malignant lesions which do not produce mucin, including CCA, may not always present with biliary dilatation. These studies should also evaluate if the thresholds shown herein will be valid when the obstruction is in different places such as seen with ampullary and hilar cholangial carcinomas. These evaluations will further support health economic evaluations (including cost-effectiveness) associated with the inclusion of MRCP+ in clinical management.

## Conclusions

In conclusion, quantitative MRCP provides imaging biomarkers that can be used to evaluate the biliary tree in a manner that can successfully discriminate benign from malignant biliary obstructions. In particular, biliary tree volume of ≥ 25 ml has clinical utility to differentiate malignant (including CCA) from benign biliary obstructions with comparable performance to experienced radiologists. Given that current pathways requires either contrast administration or ERCP, quantitative MRCP may offer an objective, non-invasive alternative to identify malignant biliary obstructions and improve patient care.

## Data Availability

The data and analytic methods used in this study remain the property of the study sponsors. All deidentified participant data may be made available to other researchers upon request following permission, investigator support and following a signed data access agreement. Requests to access the datasets should be directed to Dr Elizabeth Shumbayawonda E: Elizabeth.Shumbayawonda@perspectum.com.
