# Supplementary material for: Quantitative MRCP metrics as imaging biomarkers to differentiate benign from malignant bile duct obstructions
Source: Front Oncol. 2025 May 6;15:1576163. doi: 10.3389/fonc.2025.1576163 (PMC12088979; doi:10.3389/fonc.2025.1576163)

**Supplementary Table and Figure legends**

**Supplementary Table 1:** Routine Imaging Protocol

**Supplementary Figure 1:** Diagnostic accuracy of biliary tree volume to stratify between patients with benign or malignant biliary obstructions. The accuracy of biliary tree volume (red line) (AUC=0.82) and biliary tree volume cut-off of ≥ 25 mL (AUC = 0.78) are comparable to those of experienced radiologists (AUC ranged from 0.84 to 0.92) (p=0.223)

**Supplementary Table 1:** Routine Imaging Protocol

|  | **MR Sequences** | | | | | | | | |
| --- | --- | --- | --- | --- | --- | --- | --- | --- | --- |
| **Parameters** | **T2** | **T2** | **T2 Long TE** | **T1 IP&OP** | **T1 Dixon** | **DWI/ADC** | **MRCP** | **MRCP** | **T1 DCE*** |
| **Planes** | Coronal | Axial | Axial | Axial | Axial | Axial | Thick Slap | Thin-Slice | Axial |
| **Number of Slices** | 30 | 32 | 32 | 32 | 72 | 32 | 1 | 104 | 80 |
| **Slice Thickness (mm)** | 5 | 6 | 6 | 6 | 3 | 6 | 40 | 1 | 3 |
| **FOV (mm)** | 360 | 340 | 340 | 340 | 340 | 380 | 300 | 350 | 380 |
| **TR (ms)** | 1200 | 1200 | 1200 | 130 | 6.72 | 7000 | 3800 | 2500 | 4.48 |
| **TE (ms)** | 92 | 94 | 181 | 2.38 | 2.39 | 60 | 742 | 700 | 2.19 |
|  |  |  |  | 4.87 | 4.77 |  |  |  |  |
| **Flip angle (degrees)** | 180 | 130 | 180 | 70 | 10 |  |  | 140 | 10 |
| **Fat Suppression** | None | SPAIR | None | None |  | SPAIR |  | Fat Sat | Fat Sat |

*Performed in pre-contrast, arterial phase, venous phase, 3-min, and 5-min-post contrast

**Supplementary Figure 1:** Diagnostic accuracy of biliary tree volume to stratify between patients with benign or malignant biliary obstructions. The accuracy of biliary tree volume (red line) (AUC=0.821) and biliary tree volume cut-off of ≥ 25 mL (AUC=0.776) are comparable to those of experienced radiologists (AUC ranged from 0.838 to 0.923) (p=0.223).


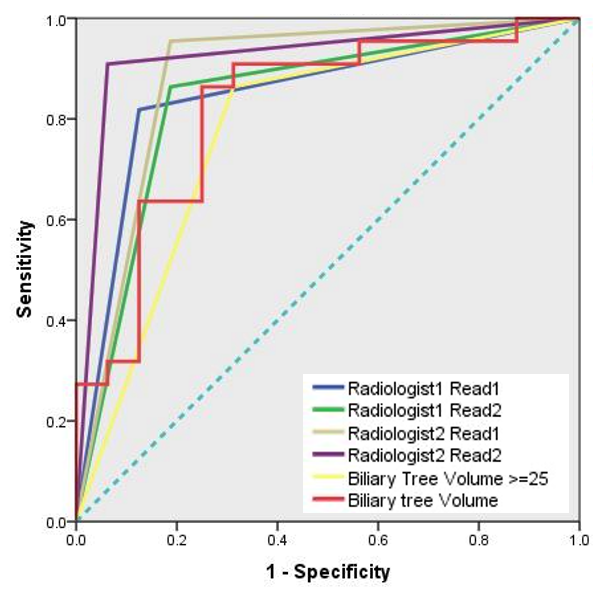

Supplement: Supplementary file 1 [file DataSheet1.docx]
